# Dietary Patterns and Associated Microbiome Changes that Promote Oncogenesis

**DOI:** 10.3389/fcell.2021.725821

**Published:** 2021-11-12

**Authors:** Shakhzada Ibragimova, Revathy Ramachandran, Fahad R. Ali, Leonard Lipovich, Samuel B. Ho

**Affiliations:** ^1^ College of Medicine, Mohammed Bin Rashid University of Medicine and Health Sciences, Dubai Healthcare City, Dubai, UAE; ^2^ Department of Medicine, Mediclinic City Hospital, Dubai Healthcare City, Dubai, UAE

**Keywords:** microbiome, dietary pattern, Western diet, plant-based diet, colorectal cancer, alcohol, oncogenesis

## Abstract

The recent increases in cancer incidences have been linked to lifestyle changes that result in obesity and metabolic syndrome. It is now evident that these trends are associated with the profound changes that occur in the intestinal microbiome, producing altered microbial population signatures that interact, directly or indirectly, with potentially pro-carcinogenic molecular pathways of transcription, proliferation, and inflammation. The effects of the entire gut microbial population on overall health are complex, but individual bacteria are known to play important and definable roles. Recent detailed examinations of a large number of subjects show a tight correlation between habitual diets, fecal microbiome signatures, and markers of metabolic health. Diets that score higher in healthfulness or diversity such as plant-based diets, have altered ratios of specific bacteria, including an increase in short-chain fatty acid producers, which in turn have been linked to improved metabolic markers and lowered cancer risk. Contrarily, numerous studies have implicated less healthy, lower-scoring diets such as the Western diet with reduced intestinal epithelial defenses and promotion of specific bacteria that affect carcinogenic pathways. In this review, we will describe how different dietary patterns affect microbial populations in the gut and illustrate the subsequent impact of bacterial products and metabolites on molecular pathways of cancer development, both locally in the gut and systemically in distant organs.

## Introduction

Cancer is one of the leading causes of mortality amongst all ages and ethnic groups worldwide. The incidence of cancer has been increasing every year, with a particularly dramatic increase in developing countries. In fact, over the last two decades, 55% of total cancer incidences were documented in developing countries. It is predicted that by the year 2050, this number will reach to 70% (Bray et al., 2018; Sung et al., 2021). With this current trend, GLOBOCAN predicts the cancer burden to rise to 27.5 million new cases per year by 2040 (Sung et al., 2021). Globally, lung, breast, and colon cancers are the most frequently occurring cancers. This recent increase in cancer incidence has been linked to changes in lifestyles that have resulted in an increase in obesity and the metabolic syndrome, which are among the leading risk factors for cancer (Hulvat, 2020).

With emerging novel technologies, there is an abundance of information and new knowledge of cancer biology each year. Among those, recent advancements in metagenomics have allowed for better characterization of the human gut microbiome diversity and its impact on the host organism’s predisposition to various cancers (Yu et al., 2017). Approximately half of the cells in the human body are those of commensal bacteria, not human cells (Sender et al., 2016). Nevertheless, the interplay between the microbiome and disease has only recently begun to be illuminated. The human gut microbiome is an extremely dynamic “organ”, with multiple factors constantly affecting the diversity and composition of this microenvironment. These factors include diet, lifestyle, drugs such as antibiotics, delivery method at childbirth and the genetic makeup of the individual (Wen and Duffy, 2017). It is now evident that diet is a major contributor to the variation in gut microbiota, producing population signatures that interact, both directly and indirectly, with molecular pathways affecting key biological processes including transcription, proliferation, and inflammation, that can have pro-carcinogenic effects (Cheung et al., 2017). Evolutionarily, the human diet has always been shaped by many different factors, including cultural, geographical, economical, and seasonal. However with the dramatic increase in the rate of globalization in recent decades, the boundaries between various dietary patterns and lifestyles have blended (Arkan, 2017). Within the context of this global trend, the broad assumption—supported by progressively emerging evidence—is that diet can make us susceptible to certain diseases through alteration of our microbiome composition.

Of late, research on the microbiome has garnered enormous attention from the scientific world, as well as from the public. Although microbiome research is still in its infancy, it has already been established that the microbiome has a direct influence on almost all the pathophysiological processes in the human body (Asnicar et al., 2021; George et al., 2021). The composition of the gut microbiome and its interaction with the cellular processes in the gut epithelium have been shown to predispose an individual to certain diseases, including but not limited to colorectal, liver, and other cancers, inflammatory bowel disease (IBD) and other autoimmune and neurologic diseases including Alzheimer’ (Tedelind et al., 2007; Nosho et al., 2016; Vogt et al., 2017). However, due to the tremendous diversity of gut microbiome, and the heterogeneity of cancer pathophysiology, the direct link between microbiome composition and cancer pathogenesis is yet to be established (Scott et al., 2019). The purpose of this review is to summarize recent research into the relationship between dietary patterns and the gut microbiome and associated metabolome, and how they directly regulate pro-carcinogenic pathways. We look at the effect of altered gut microbiome locally in the gut colonic tissue and environment and systemically, where microbiome-derived toxins and metabolites affect distant organs via portavenous and arterial circulation. For comparison, this review also focuses on the beneficial effects of a healthier, “plant-rich” diet.

## Dietary Patterns and Cancer Risk

It is challenging to define an absolute dietary pattern, particularly as most observational and research studies traditionally focus on key components of certain types of diet, excluding the overall effects and the synergy between dietary components (Klement and Pazienza, 2019). However, in the real-world, and as direct consequence of globalization making every type of diet accessible in nearly every country on-demand, people do not consume isolated products. Moreover, due to the global coverage of the food manufacturing industry, consumers in many countries are not given the information necessary to understand what the exact constituents and additives of their foods are. Today, nutritional epidemiology is trying to observe the changes in human health due to the overall dietary composition, since the predominance and the trend of specific diets is more important than consumption of certain isolated foods (Hosseinkhani et al., 2021). Researchers studying nutrition and health often use food frequency questionnaires (FFQs) to track the frequency of foods consumed over a fixed period of time. The FFQs are then evaluated using different dietary indices, that quantitatively measures an individual diet’s adherence to dietary guidelines in order to correlate a person’s diet history with various health outcomes such as obesity or biomarker concentrations (Supplementary Table S1). In this review, the term “dietary pattern”, will be used to segregate and draw the line between “Western type of diet” and “Plant-based diet”.

The dietary pattern labeled “Western diet” is characterized by relatively high fat content, particularly saturated fats, highly processed carbohydrates, and lesser amounts of fiber (Table 1). The main dietary signature of the western type of diet is the overconsumption of processed food that has undergone chemical treatment and has a high level of emulsifiers and other synthetic additives (Cordain et al., 2005). Moreover, increased consumption of refined sugar, dietary salt and animal-based products, especially red meat, are also key attributes of the Western diet, along with dramatically decreased consumption of dietary fiber (Zhong et al., 2021). The term Western diet has predominantly come from the correlation of a set of diseases that mostly occur in the western world (Cordain et al., 2005). Interestingly, the term Western diet is also used to describe a specific high-fat diet used in some animal studies, however this does not apply to this review. An increasing number of quantitative studies have demonstrated positive links between continuous overconsumption of red meat and ultra-processed food (UPF) as one of the key drivers of developing different types of tumors (Chen et al., 2020a). Adherence to the Western diet is thus considered as one of the main risk factors in developing cancer (van den Berg et al., 2021).

**TABLE 1:** Different dietary patterns and components.

Broadly classified as (in this review)	Diet name	Dietary components	Less frequent
		Frequent	
PLANT-BASED	Vegan (Craig, 2009)	Fruits and Vegetables	Dairy and eggs
		Whole Grains	Animal products
		Legumes and Beans	Fish
		Nuts and seeds	Meat products
	Vegetarian (Melina et al., 2016)	Fruits and Vegetables	Meat products
		Whole Grains	Fish
		Legumes and Beans	
		Nuts/Seeds	
		Dairy and eggs	
	Mediterranean (Di Daniele et al., 2017)	Fruits and Vegetables	High intake of red meat
		Whole Grains	Dairy
		Legumes and Beans	
		Nuts/Seeds	
		Fish	
		Unsaturated fats such as olive oil	
	DASH (Dietary Advances to Stop Hypertension) (Vogt et al., 1999)	Fruits and Vegetables	High-salt, high-sugar, highly-processed foods
		Low-fat dairy products	Refined carbohydrates
WESTERN	Omnivore (Cordain et al., 2005)	Fruits and Vegetables	
		Whole Grains	
		Fish	
		Meat	
	Western pattern diet (Clarys et al., 2013)	Red meat/Processed meat	Vegetables, Fruits
		Pre-packaged and fried foods Butter	Whole grains
		Candy and sweets	
		High-fat dairy products	
		Refined grains	
		High-fructose corn syrup drinks	

Numerous cohort and case control studies have been designed to interrogate the correlation between processed food consumption and various health outcomes. A recent cohort study, amongst the French population from 2009 to 2017, determined that the risk of developing cancer directly correlates with the increased consumption of UPF (Fiolet et al., 2018). The same French cohort study has also observed a direct association between UPF intake and weight gain, as well as increased risk of developing obesity (Beslay et al., 2020). A German population-based case-control study has established strong correlation between increased intake of not only processed meat but also red meat with increased risk of developing colorectal cancer (CRC) in mixed age groups (Carr et al., 2017). A population-based case-control study in Israel, has also established the dose-dependent correlation between the concurrent intake of UPF and smoking on one hand and the severity of colorectal neoplasia on the other, with higher intake of UPF and smoking resulting in more advanced colorectal adenomas (Fliss-Isakov et al., 2020).

A systematic review, analyzing 11 meta-analysis of the effect of red meat in development of CRC, concluded that increased intake of red meat and processed meat elevated CRC risk by 20–30% (Aykan, 2015). Another systematic review, focusing on the broader effect of UPFs on the health outcomes, pulling from 12 recent cohort studies and 8 cross-sectional studies, demonstrated significant correlation between UPF intake and increased risk of developing obesity, type II diabetes and several types of cancer (Chen et al., 2020a).

In contrast to the Western diet, a plant-based dietary pattern, which includes the vegetarian, vegan, and Mediterranean diets, features a preponderance of the dietary components that are plant-based, such as whole grains, plant-derived oils, and legumes, with significantly reduced animal and fish products, as well as minimal to no processed food (Table 1). Although, geographically plant-based diet is mostly predominant in the regions of Mediterranean basin (Klement and Pazienza, 2019), in the recent years plant-based diet is acquiring more followers around the globe due to its established protective properties (Tsvetikova and Koshel, 2020). High number of recent epidemiological studies claim protective effects of a plant-based diet from a set of non-communicable diseases (NCDs), such as the metabolic syndrome and various types of cancer, especially CRC (Pasolli et al., 2019; Alexandrov et al., 2020). Furthermore, adherence to healthier plant-based dietary patterns results in a quick shift in microbial population towards more beneficial bacteria (Alexandrov et al., 2020; Kim et al., 2020). This shift in microbial populations has multiple consequences which have been linked with improved tight-junction homeostasis of the gut epithelium layer, better immune surveillance, and control over inflammatory processes (Tsvetikova and Koshel, 2020; Asnicar et al., 2021) as we will explore later.

The relationship between dietary patterns and the microbiome has been investigated by many studies, with the significant recent addition of the extensive Arivale/Institute for Systems Biology study (Manor et al., 2020) and the international multicenter PREDICT-1 study (Asnicar et al., 2021). The Arivale study analysed 3,409 individuals enrolled in a wellness program with extensive characterization of metabolic markers, lifestyle, diet, and stool microbiome 16S amplicon sequencing. They found that specific microbial populations with increased diversity were associated with improved cardiometabolic markers and plant-based dietary patterns (health-related group). Conversely, they found that specific microbial populations with less diversity were associated with worse cardiometabolic and lifestyle markers (disease-related group). The health related group had increased genera *Coprococcus, Lachnospira, Faecalibacterium*, and unclassified genera from the Ruminococcaceae and Clostridiales family/order. The disease-related group had increased abundance of the genera *Bacteroides, Ruminococcus, Sutterella, Bilophila, Acidaminococcus*, and *Megasphaera*. PREDICT-1 investigators analyzed stool microbiome metagenomic sequencing data from 1,098 individuals from the UK and United States, and correlated the results with demographic variables, detailed dietary logs, and cardiometabolic blood markers (Asnicar et al., 2021). Major findings from the study included that the intrasample alpha diversity, or an estimate of the total number or richness of bacterial species in a sample, significantly correlated with 56 of 295 tested correlations with personal characteristics, habitual diet and metabolic markers. Microbiome species richness was positively correlated with favorable high-density lipoprotein levels, whereas body-mass index (BMI), visceral fat, and probability of fatty liver were inversely correlated with species richness. Data from individual food diaries were evaluated using validated dietary indices such as the alternate Mediterranean diet score (aMED), Healthy Eating Index (HEI) and the Plant-based Dietary Indices (PDI) that have previously been shown to correlate with reduced risk of chronic diseases (Supplementary Table S1). These indices showed a tight correlation with microbial composition, demonstrating how habitual diets influence the microbiome. Out of the 30 bacterial species that showed the strongest overall correlation with markers of nutritional and cardiometabolic health, 15 species were positively associated with healthy plant-based diets and negatively associated with visceral fat, liver fat probability, and high-risk metabolic markers. These included *F. prausnitzii, Proventella copri, Roseburia*, *Oscillibacter*, and several *Firmicutes* species. Conversely, the other 15 bacterial species were negatively associated with healthy diets, and positively associated with increased visceral fat, liver fat probability, and high-risk metabolic markers. These included *Clostridia* species, *R. gnavus*, and *F. plautil*. The repertoire of 30 bacterial species represents a novel composite quantitative marker of the link between dietary patterns and cardiometabolic health, and broadly support a dichotomous clinically relevant separation of healthy “plant-based” and less healthy “western, high fat” dietary patterns with the associated “healthy” microbiome or eubiosis and the “unhealthy” microbiome or dysbiosis (Table 2) (Asnicar et al., 2021). Furthermore, diet and gut microbiome has been directly linked with the circulating metabolome of human serum (Bar et al., 2020). Bar et al. found that over 50% of the observed variance in 1,251 human serum metabolites was explained by diet and microbiome variables, in a study of 491 subjects who were carefully phenotyped according to genetics, gut microbiome, diet and lifestyle measures (Bar et al., 2020). Finally, a recent longitudinal study of 307 well characterized subjects correlated adherence to the plant-based Mediterranean diet with specific microbial and functional patterns which in turn correlated with favorable cardiometabolic markers (lipids, c-reactive protein, and hemoglobin A1C) (Wang et al., 2021). These studies strongly suggest the mechanisms whereby adherence to a Mediterranean diet results in reduced cardiovascular, metabolic, and cancer related outcomes demonstrated in the PREDIMED randomized clinical trial (Estruch et al., 2018; Toledo et al., 2015). More prospective randomized intervention trials of dietary components and specific bacterial communities are needed to further test the link between dietary composition, microbiome structure/function parameters, and cardiometabolic and oncogenic outcomes.

**TABLE 2:** Microbial signatures of the gut microbiome, in respect to Western dietary (WD) pattern and plant-based (PD) dietary pattern (N/I non-identified).

	Bacterial signature of WD microbiome (De Filippis et al., 2016; Asnicar et al., 2021)	Function	Bacterial signature of PD microbiome (De Filippis et al., 2016; Asnicar et al., 2021)	Function
Increased Population	Clostridium bolteae	Increase cardiometabolic risk	Faecalibacterium prausnitzii	Butyrate production
	Atopobium parvulum	Hydrogen sulfide production	Roseburia intestinalis	Butyrate production
	Actinomycosis odontolyticus	Hydrogen sulfide production	Akkermansia muciniphila	Enhancement of mucin production
	Bilophila wadsworthia	Secondary bile acids production	Prevotella copri	Glucose homeostasis, improvement in postprandial glucose responses
	Streptococcus bovis/gallolyticus	Nitric oxide production	Roseburia hominis	Butyrate production
	Clostridium saccharolyticum	Increase in cardiometabolic risk	Agathobaculum butyriciproducens	Butyrate production
	Clostridium innocuum	Increase cardiometabolic risk	Anaerostipes hadrus	Butyrate production
	Clostridium symbiosum	Increase cardiometabolic risk	Firmicutes bacterium	Postprandial lipoprotein remodeling
	Clostridium spiroforme	Increase cardiometabolic risk	Haemophilus parainfluenzae	Reduces GlycA levels, systemic inflammation, cardiometabolic risks
	Clostridium leptum	Increase cardiometabolic risk	Eubacterium eligens	N/I
	Flavonifractor plautii	Increase cardiometabolic risk	Lawsonibacter asaccharolyticus	Butyrate production
	Ruthenibacterium lactatiformans	N/I	Oscillibacter sp	N/I
	Escherichia coli	N/I	Streptococcus thermophilus	Probiotic
	Collinsella intestinalis	N/I	Bifidobacterium animalis	Probiotic
	Eggerthella lenta	N/I		
	Anaerotruncus colihomini	N/I		
	Clostridium spiroforme	Increase cardiometabolic risk		
	Ruminococcus gnavus	Increase cardiometabolic risk		
Decreased Population	Lactobacillus acidophilus (Prebiotic bacteria)	Reduces nitroreductase activity		
	Prevotella copri	Glucose homeostasis, improved postprandial glucose responses		
	SCFAs producers	SCFA production, immune homeostasis		

## Molecular Mechanisms of the Role of Plant-Based Diet in Cancer Protection

**FIGURE 1:**
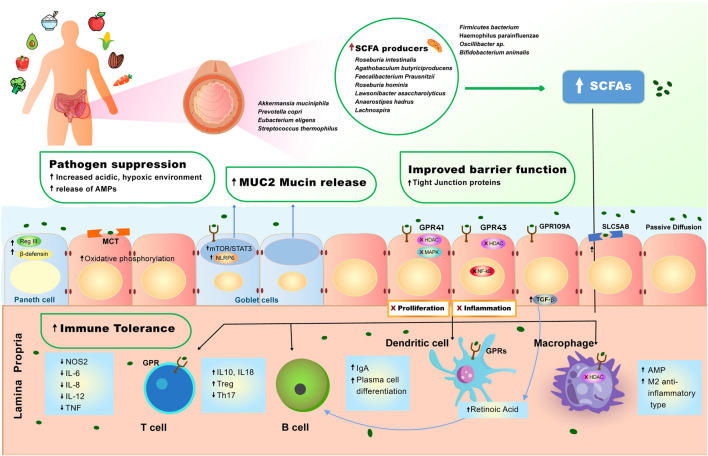
Effect of plant-diet on the gut microbiome and intestinal homeostasis. A diet rich in plant-based foods results in high abundance of short-chain fatty acid (SCFA) producing bacteria in the gut lumen. High production of SCFAs by gut microbiome is strongly associated with pathogen suppression, mucus production, improved barrier function and immune tolerance (see *Molecular Mechanisms of the Role of Plant-Based Diet in Cancer Protection*). SCFAs induce a set of anti-inflammatory events via interaction with GPR41, GPR43, GPR109A receptors and MCT and SLC5A8 transporters that are expressed on the surface of intestinal epithelial cells and immune cells. 1) SCFAs bind GPR109A on the epithelial cell which leads to increase in TGF- β production, enhancing the differentiation of Treg cells, which leads to increase of immune tolerance in the gut. 2) SCFAs also bind to GPR43 inhibiting HDAC and NF- κB, resulting in decreased inflammation. 3) SCFAs bind to GPR41, inhibiting HDAC and MAPK signaling pathway, resulting in inhibition of proliferation. 4) SCFAs cross the epithelial cell barrier and reach lamina propria, where they induce cascade of anti-inflammatory reactions, regulating immune homeostasis in the lower bowel. In the lamina propria, SCFAs bind to macrophage (MQ cell) inhibiting HDAC, facilitating polarization of macrophages to M2-anti-inflammatory type, and increasing production of antimicrobial peptides (AMPs). 5) SCFAs bind to dendritic (DC) cell enhancing retinoic acid production, further facilitating production immunoglobulin A (IgA) and differentiation of Tregs. 6) SCFAs induce production of TGF- β and retinoic acid, causing B cells to stimulate production of IgA and facilitate plasma B cell differentiation. 7) SCFAs bind to GPR on T cells, initiating the production of IL-10, which increases Tregs pool, and inhibits expansion of T helper (Th) 17.

The gut microbiome and the human intestinal immune system have co-evolved over evolutionary time to stay in balance and to regulate each other, the ideal status quo that corresponds to a healthy state. This balance is composed of four elements (Figure 1). First, the bacteria pathogens are suppressed and are kept compartmentalized from the intestinal epithelium by a thick mucus layer, the release of antimicrobial peptides (RegIIIg) and secreted IgA to protect the epithelial surfaces from invasion, and the presence of intraepithelial immune cells and neutrophils that can migrate into the intestinal lumen. Second, the intestinal epithelium has tight junctions that form the epithelial barrier that are strengthened by luminal metabolites (Biragyn and Ferrucci, 2018). Third, inputs from luminal antigens regulate intestinal macrophages to become hyporesponsive and exhibit tolerance, producing IL-10 and less inflammatory cytokines (Round and Mazmanian, 2010; Vinolo et al., 2011). Fourth, there is active suppression of microbe reacting effector T-cells by Foxp3b Treg cells and Roc-3-Tr1 cells via IL-10; and intraepithelial immune cells use MHC to present bacterial antigens and down regulates reactive CD4b T cells (Geuking et al., 2011; Zhang et al., 2019).

Maintenance of a healthy balance for the intestinal barrier and immune homeostasis depends on a balance of beneficial bacteria and dietary nutrients, especially dietary fiber. Fiber is a major component of the plant-based diet and is an indigestible carbohydrate for the mammalian gastrointestinal tract, consequently certain bacterial phyla in the gut are responsible for fiber fermentation and production of short-chain fatty acids (SCFAs) (Wilson et al., 2020), which is a key metabolite in a continuous dialogue between the host and the gut microbiome.

Homeostasis of the mucus layer is a key component in intestinal health (Cai et al., 2020; Paone and Cani, 2020). Mucus serves as a mechanical and chemical shield, protecting epithelial cells from the pathogenic attacks. Along with Goblet cells, the production of mucus is maintained by the commensal bacteria in the gut microbiome (Paone and Cani, 2020). Numerous studies have observed that high-fat Western diet promotes the deterioration of intestinal mucus layer, whereas plant-based diet enhances mucus layer thickness. One result of low fiber diets is the reduced delivery of fiber to these bacteria, resulting in a switch to metabolizing endogenous carbohydrates present on intestinal mucin glycoproteins. This results in a reduction in the quality of the protective intestinal mucous coat (Png et al., 2010; Ijssennagger et al., 2015). This was shown experimentally by Desai et al. using a gnotobiotic mice colonized with a synthetic human gut microbiota (Desai et al., 2016). When these mice were fed a fiber deficient diet, mucin degrading bacteria levels increased and the susceptibility to enteric pathogens increased (Desai et al., 2016). These data indicate the importance of adequate dietary fiber in maintaining epithelial barrier protection.

Due to the absence of mammalian enzymes that can degrade carbohydrates, especially resistant starch, certain *Firmicutes* and *Bacteroides* species are able to ferment indigestible carbohydrates leading to the production of SCFAs (Biragyn and Ferrucci, 2018). Evidently the predominant majority of SCFAs produced are acetate, propionate, and butyrate.

SCFAs are extremely bio-active molecules, primarily acting through interaction with G-protein coupled receptors: GPR41, GPR43, and GPR109A and by direct inhibition of histone deacetylase (HDAC). Uptake of SCFAs in colon epithelial cells occurs by multiple mechanisms. These include passive diffusion, specific monocarboxylate transporters (MCT), as well as through SLC5A8 receptors (Ulven, 2012). Increased production of SCFAs by gut microbiome is strongly associated with the improvement of barrier junctions, increase in protective mucosal layer, increase in immune tolerance and suppression of intestinal inflammation (Smith et al., 2013; Schwiertz et al., 2010). SCFAs are produced in the gut lumen where they interact with intestinal epithelial cells, however SCFAs are also able to cross the epithelial layer and reach the lamina propria, where they can then interact with the set of immune cells, and also enter into the systemic circulation (Figure 1) (Blacher et al., 2017; Zhang et al., 2019).

A recent study has shown that mice fed on low fiber intake resulted in depletion of butyrate production, which in turn directly caused disruption of the gut microbial diversity, leading to systemic inflammation and mortality from necrotizing pancreatitis. The mortality rate in these mice significantly decreases upon oral and systemic introduction of butyrate (van den Berg et al., 2021). Immune regulation and maintenance of anti-inflammatory environment in the gut lumen is extremely convoluted process, and its disturbance leads to accumulated mechanisms that may promote oncogenesis. It is now established that propionate and butyrate bind to GPR43 on the surface of Foxp3+ expressing Treg cell, and facilitate the differentiation of Treg cells as well as elevate the production of IL-10, hence controlling and decreasing level of intestinal inflammation, and increasing immune tolerance at a large scale (Smith et al., 2013; Mandaliya et al., 2021). Another mechanism by which SCFAs regulate Treg cell differentiation is through the production of TGF-β by the epithelial cells (Basson et al., 2016).

Similarly to butyrate, propionate acts as a ligand for SCFA-sensing receptors GPR43, GPR41, and GPR109A (Furusawa et al., 2015), inhibiting the expression of pro-inflammatory cytokines such as IL-6, IL-8, and TNF. Propionate inhibits the MAPK signaling pathway and prevents proliferation of CRC cells (Davido et al., 2001), while acetate dramatically reduces production of pro-inflammatory agents via inhibition of NF-κB signaling pathway in CRC cells, inhibiting the cancer progression. Thus, depletion of SCFAs can lead to colonic and extraintestinal inflammation, resulting in unfavorable outcomes like inflammatory bowel disease (IBD) and CRC (Davido et al., 2001). Mandaliya et al. introduced butyrate and propionate to diabetic mice that were fed a high fat diet (HFD), observing both T cell polarization and inhibition of pro-inflammatory IL-6 cytokine production dramatically minimizing inflammation grade in the gut (Mandaliya et al., 2021). A single-blind pilot study compared the contribution of dietary fiber towards SCFAs concentration and Treg cell population, showing the group with higher intake of dietary fiber demonstrated a dramatic increase in total CD4+T cells and Tregs. Moreover, upon activation of its receptor, GRP109A, butyrate can inhibit several key pro-inflammatory pathways that are highly involved in CRC, such as protein kinase B or Akt (PKB/Akt) and NF-κB signaling pathways (Chen et al., 2018).

SCFAs regulate epigenetic changes via their ability to inhibit histone deacetylase (HDAC). HDAC inhibition regulates the levels of Treg cells in the colon, though the mechanism is not fully understood (Verma and Shukla, 2013). Through HDAC inhibition, butyrate also initiates polarization of macrophages to M2 phenotype, hence leading to a decrease in inflammatory ability of macrophages cells (Li et al., 2019a). Butyrate also acts on the population of intestinal macrophages to promote production of anti-inflammatory IL-10 and through the inhibition of HDAC, initiates the production of antimicrobial peptides (AMPs), boosting pathogenic clearance in the intestine (Mohammadi et al., 2018). Moreover, HDAC inhibition in macrophages leads to the dramatic decrease in production of pro-inflammatory cytokines such as, IL-6, IL-12, nitric oxide (NO), and TNF (Scott et al., 2018). The inhibition of HDAC activity by propionate, reduced the level the tumorigenic lesions in the colon (Casanova et al., 2018).

Furthermore, interaction between SCFAs and intestinal epithelial cells leads to the elevated secretion of NLRP3 inflammasome that further results in increased secretion of IL-18, hence facilitating the improvement of tight junction’s homeostasis (Macia et al., 2015; Nowarski et al., 2015). Moreover, SCFA-receptors are also present on Paneth, Goblet and L cells, hence binding of SCFAs to these receptors’ triggers production of molecules of defending nature. When SCFAs bind GPCR on Goblet cells, it triggers the activation of NLRP6, as well as mTOR/STAT3 signaling pathway to increase mucus production in the gut lumen (Wlodarska et al., 2014; Li et al., 2019b). Simultaneously, butyrate acts via Paneth cell GRP43 receptor resulting in the production of key anti-microbial peptides, such as RegIIIγ and β-defensin (Birchenough et al., 2016). These two cascades of reactions, also lead to an improved barrier junction, as well as elevated innate response to the continuous flow of pathogens. Interestingly, enteroendocrine L cells, that are part of the colonic epithelium and express SCFA receptor on their surface, upon the interaction with acetate and butyrate, produce glucagon-like peptide-1 (GLP-1) and fasting peptide YY (PYY) peptides (Brooks et al., 2017; Zhao et al., 2018) These peptides are thought to increase energy intake while decreasing appetite, hence these peptide are involved in the gut-brain axis and are potential therapeutic agents in treating conditions like obesity. In summary, the majority of research studies support that SCFA derived from dietary fiber plays a key role in epithelial defenses and immune regulation in the colon, however further research is essential to better understand benefit-based stratification amongst various dietary fiber types. For instance, a recent study by Singh et al. has demonstrated that diet rich in soluble inulin fiber provoked icteric hepatocellular carcinoma (HCC) in dysbiosis mice models (Singh et al., 2018).

## Western Dietary Pattern—Local Pathophysiologic and Molecular Effects

Direct effects of dietary constituents, microbiota, and microbial products are thought to play a causative role in colorectal carcinogenesis though multiple mechanisms, including genotoxic, inflammatory, immune mediated, and metabolic (Scott et al., 2019) In addition to being associated with a distinct microbial signature, western-type diets are characterized by certain dietary constituents (N-nitroso compounds, heterocyclic amines, and heme) and increased secondary bile acids and other metabolic products derived from enriched bacterial species that can directly promote a local pro-inflammatory and pro-carcinogenic environment in the colon. In addition, the lack of dietary fiber in western-type diets results in metabolic shifts that impact epithelial defense against inflammation (Bhaskaran et al., 2018).

The relative increase in specific bacterial genus and species in the microbiome found in patients with pre-cancerous adenomas and CRC has fueled investigations into the possible direct pro-carcinogenic or pro-inflammatory effects of these bacteria (Mima et al., 2016a; Yu et al., 2018). Initial studies focused on comparing microbiome and metabolome changes in patients with various stages of colorectal neoplasia. Yu et al. used shotgun metagenomic sequencing and identified 20 microbial gene markers that were significantly associated with CRC (Yu et al., 2017). A co-occurrence network, generated from the relative abundance of 20 bacterial species was significantly associated with CRC. Wirbel et al. conducted a meta-analysis of 8 geographically diverse fecal shotgun metagenomic studies of CRC patients (n = 768). They found a core set of 29 species that were enriched in CRC metagenomes. Functional characteristics of these core species indicated enrichment of protein and mucin catabolism genes and elevated production of secondary bile acids, likely reflecting diets high in fat and meat nutrients (Wirbel et al., 2019). Furthermore, Ng et al. used metagenomic sequencing to describe a specific virome and mycobiome signatures associated with CRC (Ng et al., 2019).

Other studies have interrogated the temporal framework changes in the microbiome during adenoma-carcinoma development sequence. Fecal samples from patients with CRC, advanced adenomas, non-advanced polyps, and normal subjects were studied to represent different stages of neoplastic evolution. Nakatsu et al. found significant differences in the mucosal bacterial communities found in normal mucosa, adenoma, and carcinoma samples (Nakatsu et al., 2015). Zhang et al. showed that the relative abundance of 24 bacterial species significantly changed in fecal samples between normal, non-adenomatous polyps, adenomas and carcinoma groups of patients, with relatively higher amounts of *Fusobacterium nucleatum* and pro-inflammatory periodontal bacteria, along with lower amounts of beneficial short-chain fatty acid (SCFA) producers in the CRC groups compared with the other groups (Zhang et al., 2018). This correlated with a trend for increasing C-reactive protein and STNFR-II in the adenoma and CRC groups (Zhang et al., 2018). Similarly, Hale et al. found modest but significant changes in the fecal 16S rRNA gene characterization of microbiota of 547 adenoma patients compared with 233 patients without adenomas (Hale et al., 2017). Taxa that were more abundant in patients with adenoma included *Bilophila*, *Desulfovibrio*, proinflammatory *Mogibacterium*, and Bacteroidetes species; whereas taxa that were increased in patients without adenomas included *Veillonella*, Firmicutes, Clostridia and *Actinobacteria*.

Kim et al. expanded these observations to focus on the differences in the fecal microbiome and metabolome signatures of 102 patients with advanced adenomas compared with 102 matched healthy controls without polyps and with 36 patients with CRC, adjusting for sex and age in the groups. They found that several bioactive lipid pathways were significantly associated with the adenoma group, including polyunsaturated fatty acids, secondary bile acid pathways, endocannabinoid metabolism, and sphingolipid pathways (Kim et al., 2020). The relative amounts of these lipids were not quantified, which could determine the pro-vs anti-inflammatory effects of these lipids. These metabolomic pathways were correlated with genus-level microbiome sequencing, and they found positive correlations with *Bacteroides* and 10 or more sub pathways, whereas four genera from *Firmicutes* and one from *Actinobacteria* showed negative correlation with these pathways (Kim et al., 2020). The authors stated that the changes observed in the metabolic pathways are too small to be useful as diagnostic markers of adenomas, however these observations illustrate the possible biological pathways that are early events in the adenoma-carcinoma pathway. Overall, these studies indicate that there are specific fecal and mucosal microbiome signatures associated with the development of colonic neoplasia.

**FIGURE 2:**
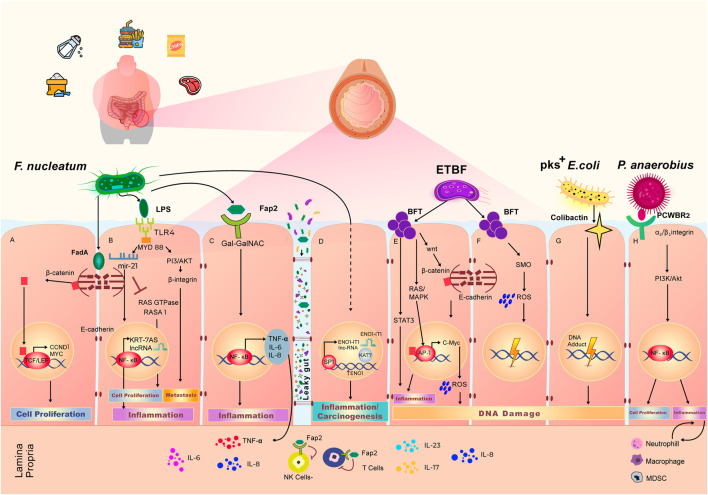
Effect of Western diet on the gut microbiome and local pro-carcinogenic pathways. Adherence to Western diet results in the high abundance of *Fusobacterium* *nucleatum*, Enterotoxigenic *Bacteroides fragilis* (ETBF), pks_+_ *Escherichia* *coli* and *Peptostreptococcus* *anaerobius*. Overpopulation of these bacteria in the gut microbiome leads to the increased cell proliferation, elevated grade of inflammation and accumulation of DNA damage, eventually resulting in an environment favorable to initiation or progression of colorectal cancer. **(A)** *F. nucleatum* secretes FadA toxin, which binds the E-cadherin complex on the wall of intestinal epithelial cells. Upon interaction with FadA, E-cadherin undergoes phosphorylation, which lead to cleavage and subsequent translocation of the β-Catenin component of the complex to the nucleus. β-Catenin binds with TCF/LEF transcription complex, triggering the expression of CCND1 and MYC, that govern cell proliferation. **(B)** LPS is a polysaccharide on the cell wall of *F. nucleatum*, that binds the TLR4 receptor which are expressed on the intestinal epithelial cell membrane. LPS-TLR4 complex activates MYD88 adaptor that results in inhibition of a Ras GTPase—RASA1. The inhibition of RASA1 leads to unwanted cell proliferation. LPS-TLR4-MYD88 complex also induces the production of microRNA miR 21, that in turn activates NF-κB transcription factor and leads to the production of pro-inflammatory agents including KRT-7AS long non-coding RNA. **(C)** Upon binding of microbial protein Fap2 with cell membrane receptor, surface expressed polysaccharide Gal-Gal-NAc modulates the activation of NF-κB transcription factor that leads to the increase in intestinal inflammation through the production of TNF-α, IL-6, and IL-8. Increased intestinal permeability also, known as “Leaky gut” phenomena, allows the bi-directional passage of bacteria and their metabolites, as well as immune inflammatory agents. Through the leaky gut, Fap2 is able to reach lamina propria and diminish the activity of Natural killer (NK) cells and T cells. **(D)** *F. nucleatum* interacts with intestinal epithelial cells to stimulate the production of long non-coding RNA (lncRNA) ENO1-IT1 via SP1 transcription factor. Production of ENO1-IT1 increases concurrently the expression of ENO1. This cascade of reactions leads to the increased level of carcinogenesis and inflammation. **(E)** ETBF produces BFT toxin, that interacts with epithelial cells to trigger intestinal inflammation via activation of STAT3 signaling pathway. BFT activates RAS/MAPK pathways to further initiate AP1 and start the production of IL-8 resulting in intestinal inflammation. BFT stimulates the translocation of β-Catenin to the nucleus initiating expression of C-Myc and production of reactive oxygen species (ROS), leading to DNA damage. **(F)** BFT also induce the production of spermine oxidase (SMO) and SMO-dependent ROS production, resulting in DNA damage. **(G)** Colibactin is secreted by of pks_+_ *E. coli*. Interaction of colibactin with epithelial cells results in accumulation of DNA adducts and further DNA damage. **(H)** *P. anaerobius* surface protein PCWBR2 binds to α_2_/β_1_ integrin on the epithelial cells to initiate cell proliferation via activation of PI3K/Akt signaling pathway and NF-κB transcription factor. This interaction also leads to expansion of neutrophils, macrophages and myeloid derived suppressor cells driving chronic inflammation and tumor progression.

Directly supporting the direct carcinogenic effect of the gut microbiome is the pivotal observation that fecal samples from CRC patients promote intestinal tumorigenesis in colon carcinogenic mouse models induced by azoxymethane (Wong et al., 2017). To determine the specific mechanisms that can explain this, potentially pathogenic bacteria found to be enriched in colorectal adenomas and carcinomas have been investigated to determine if they have direct carcinogenic effects in the colon (Figure 2). These include *F. nucleatum*, Enterotoxigenic *Bacteroides fragilis* (ETBF), *pks* ^*+*^ *Escherichia coli*, and *Peptostreptococcus anaerobius* (Figure 2) (Dai et al., 2018; Haghi et al., 2019).

Although the review is focused on the microbial composition in the gut lumen, it is important to highlight arising areas of mycobiome and virome and how much they contribute to oncogenesis. Both fungal and viral composition in the gut, has been shown to be altered in patients with cancer in comparison to healthy individuals (Vallianou et al., 2021). It has also been found that CRC patients show virome dysbiosis, however more studies are required in order to fully elucidate the contribution of eukaryotic viruses in cancer development (Massimino et al., 2021).

### Fusobacterium Nucleatum

*F. nucleatum* is a Gram-negative commensal anaerobe that in the past decade has drawn a lot of attention due to its strong association with the development of colorectal neoplasia (Abu-Ghazaleh et al., 2021). There are numerous studies linking *F. nucleatum* overpopulation in the gut microbiome to local and distant cancers in the human body. Recent population-based studies have demonstrated overpopulation and presence of *F. nucleatum* in biopsies of colorectal adenomas, and patient stool screening in comparison to the healthy individuals (Mima et al., 2016b; Mehta et al., 2017). To understand how *F. nucleatum* potentially causes carcinogenesis both locally in the colon and systemically, it is important to establish the interaction of metabolites produced by *F. nucleatum* and the cells of the colonic epithelium.

FadA is one of the virulence toxins produced by *F. nucleatum* in the gut microbiome, and it plays a crucial role in initiating carcinogenic processes in the colonic epithelium. The FadA protein has a helical form which helps it to bind to the extracellular domain of E-cadherin, an epithelial cell adhesion protein E-cadherin is internalized upon interacting with FadA, and immediately phosphorylated, which results in the release and accumulation of β-catenin molecules in the cytoplasm. β-catenin which is then subsequently translocate to the nucleus interacts with T-cell factor and lymphoid enhancer factor (TCF/LEF)-family transcription factors (Kostic et al., 2013; Rubinstein et al., 2013), initiating transcription of a set of pro-inflammatory, proliferative genes and oncogenes, such as CCND1 and MYC (Abu-Ghazaleh et al., 2021). Hence, in a situation with continuous production of FadA, the transcription machinery constantly drives a set of oncogenes along with pro-inflammatory genes, resulting in proliferation of CRC cell and favorable tumor microenvironment.

However, not all CRC cells have E-cadherin on their adherent tight junctions, therefore, not all cancer progression and inflammation events are triggered via FadA activity (Rubinstein et al., 2013; Gholizadeh et al., 2017). Accordingly, another *F. nucleatum* metabolite, Fap2, has been identified to be elevated in CRC patients. A dual mechanism of Fap2 activity leads to both immune suppression and establishment of tumor microenvironment (Abed et al., 2016; Hashemi Goradel et al., 2019). A host polysaccharide Gal-Gal-NAc, was identified to be predominant on all the types of CRC cells. Gal-Gal-NAc acts as a polysaccharide modulator for Fap2, and upon this interaction, Fap2 activates NF-κB signaling pathway that result in overproduction of TNF-α, IL-6, and IL-8 (Abed et al., 2016; Abed et al., 2017; Yachida et al., 2019). These immune chemokines are extremely pro-inflammatory, hence continuous enrichment of CRC cells via Fap2 leads to chronic inflammation in the colon and enhancement of the favorable tumor microenvironment. Moreover, Fap2 binds to the TIGIT receptor on the Natural Killer (NK) cells and T-cells, inhibiting immune T cells polarization, leading cancer cells to escape from immune surveillance (Gur et al., 2015; Mima et al., 2015).

Several studies have shown that *F. nucleatum* -infected CRCs have a higher rate of proliferation. Lipopolysaccharide (LPS) is an antigen found on the cell-wall of *F. nucleatum*. This key proinflammatory, immunogenic bacterial metabolite, a well-known inducer of the Toll-like Receptor (TLR) and NF-κB pathways that lead to inflammation and oncogenic changes both locally and systemically (Salcedo et al., 2010; Yang et al., 2017a). Activated NF-κB transcription factor, results in activation of MYD88 signaling pathway and promotes an increased expression of the microRNA 21 (miR-21), which in turn inhibits the activity of RAS GTPase—RASA1 (Ciesielska et al., 2021). miR-21 was found to significantly downregulate the production of RASA1, a member of RAS GTPases family that plays a key role in inactivating set of oncoproteins such as RAS (Salcedo et al., 2010). When RASA1 is downregulated, the MAPK signaling pathways is further activated and results in CRC cell proliferation and eventual metastasis. Moreover, apart from inhibiting the production of RASA1, miR21 also downregulates Pdcd4, which is a key tumor suppressor (Yang et al., 2017a).

### Escherichia Coli

*Escherichia coli* is a symbiotic and commensal bacterium, widely occurring in oral, vaginal and intestinal microflora. Certain strains of *E. coli* can induce carcinogenic changes on the cellular level (Arthur et al., 2012). An *E. coli* strain from the B2 phylogroup that possesses polyketide synthase island (pks^+^), was found to colonizes a healthy gut microbiome in response to a shift and continuous adherence to a Western dietary pattern. Recent studies have confirmed an increased level of *pks* ^*+*^ *E. coli* strain in patients with advanced CRC (Kohoutova et al., 2014). *pks* ^*+*^ *E. coli* produces colibactin, an extremely virulent secondary genotoxin. Multiple *in vivo* studies have confirmed that colibactin introduces DNA double-strand breaks leading to genomic instability and thereby considerably elevates the risk of acquiring further mutations (Cougnoux et al., 2014; Wilson et al., 2019).

Although colonic inflammation is known to be one the key risk factors in developing CRC, and bacterial toxins drive continuous pro-inflammatory agents in the gut, there are also numerous mutational signatures that distinguish CRC patients, that are not related to the inflammatory pool. Distinctive mutational signatures were identified in 5,876 cancer patients, the majority of them with CRC (Alexandrov et al., 2020). A recent study by Pleguezuelos-Manzano et al., exposed human intestinal organoids to the genotoxic *pks* ^*+*^ *E. coli* via luminal injection for 5 months, comparing it to organoids injected with isogenic *pks* ^*-*^ mutant bacteria and then performed whole genome sequencing. They found that organoids injected with genotoxic *pks + E. coli* resulted in the same subset of mutational signatures that had been deduced from a cohort of CRC patients (Pleguezuelos-Manzano et al., 2020).

Mechanistically, colibactin induces damage due to the presence of a cyclopropane ring within its structure. Wilson et al. have demonstrated *in vivo* evidence of colibactin activity causing DNA adducts and alkylation, leading to eventual DNA damage. The study demonstrated the strong link between colibactin structure and ability to cause DNA double stranded break, through creating DNA interstand cross-link, leading to genomic instability, and further accumulation of distinct mutation leading to CRC (Wilson et al., 2019; Xue et al., 2019).

### Enterotoxigenic *Bacteroides fragilis*

*B. fragilis* is a commensal Gram-negative anaerobe, that exists in a symbiotic fashion with the host organism (Gagnaire et al., 2017). Recent studies have strongly linked adherence to the HFD Western diet to the elevated population of *Bacteroides fragilis* in the gut microbiome (Abu-Ghazaleh et al., 2021). Due to the sharp increase in CRC cases worldwide, numerous studies have been looking at the interaction of *B. fragilis* with the colonic epithelium. The colonization of colonic epithelium with *B. fragilis* is one of the key signatures of the microbiome in CRC patients. Although the causality of the events is yet to be fully determined, recent population-based studies have shown increased level of *B. fragilis* strains in patients with inflammatory bowel disease (IBD) and colitis (Dejea et al., 2018; Rashidan et al., 2018). *B. fragilis* normally comprises up to 2% of total microbiome volume (Wu et al., 2007), but only a certain strain, Enterotoxigenic *Bacteroides fragilis* (ETBF), is associated with the development of CRC and can be characterized as carcinogenic (Liu et al., 2020; Mohseni et al., 2020). ETBF releases the zinc-metalloprotease *B. fragilis* toxin (BFT), that binds a receptor on the colonocyte and induces favorable conditions for CRC progression. This occurs by establishing chronic inflammatory microenvironment with activating a set of oncogenes and initiating production of reactive oxygen species (ROS) (Hernández-Luna et al., 2019). Upon interaction between BFT and epithelial cell receptor, β-catenin molecule dissociates from the E-cadherin complex and travels to the nucleus (Chung et al., 2018). Abundant of unphosphorylated β-catenin in the nucleus initiates the NF-ĸB/AP1 transcription machinery, causing the overexpression of pro-inflammatory cytokine like IL-8 and oncogenes such as C-MYC (Cheng et al., 2020). Moreover, BFT triggers the STAT3 signaling pathway, giving rise to the continuous production of IL-17 and IL-23, significantly increasing the extent of inflammation locally in the gut (Thiele Orberg et al., 2017; Chung et al., 2018). This continuous production of BFT promotes proliferation of CRC cells and maintains chronic inflammation at the sites of colonic epithelium. Furthermore, ETBF infected colonic cells produce reactive oxygen species (ROS), leading to the progressive genomic instability, exponentially elevating risk of acquiring new mutations, and development of CRC (Cheng et al., 2020).

### Peptostreptococcus anaerobius

Another example of a potentially pathogenic species enriched in CRC samples is *Peptostreptococcus anaerobius.* This species have been shown in cell culture to have direct inflammatory and pro-oncogenic impacts by binding to integrin α_2_/β_1_ integrin receptors on cell surfaces, activating PI3K, Akt, and NF-κB to enhance proliferation, proinflammatory cytokines, and T cell suppression (Long et al., 2019).

## Western Dietary Pattern—Systemic Pathophysiologic Effects

**FIGURE 3:**
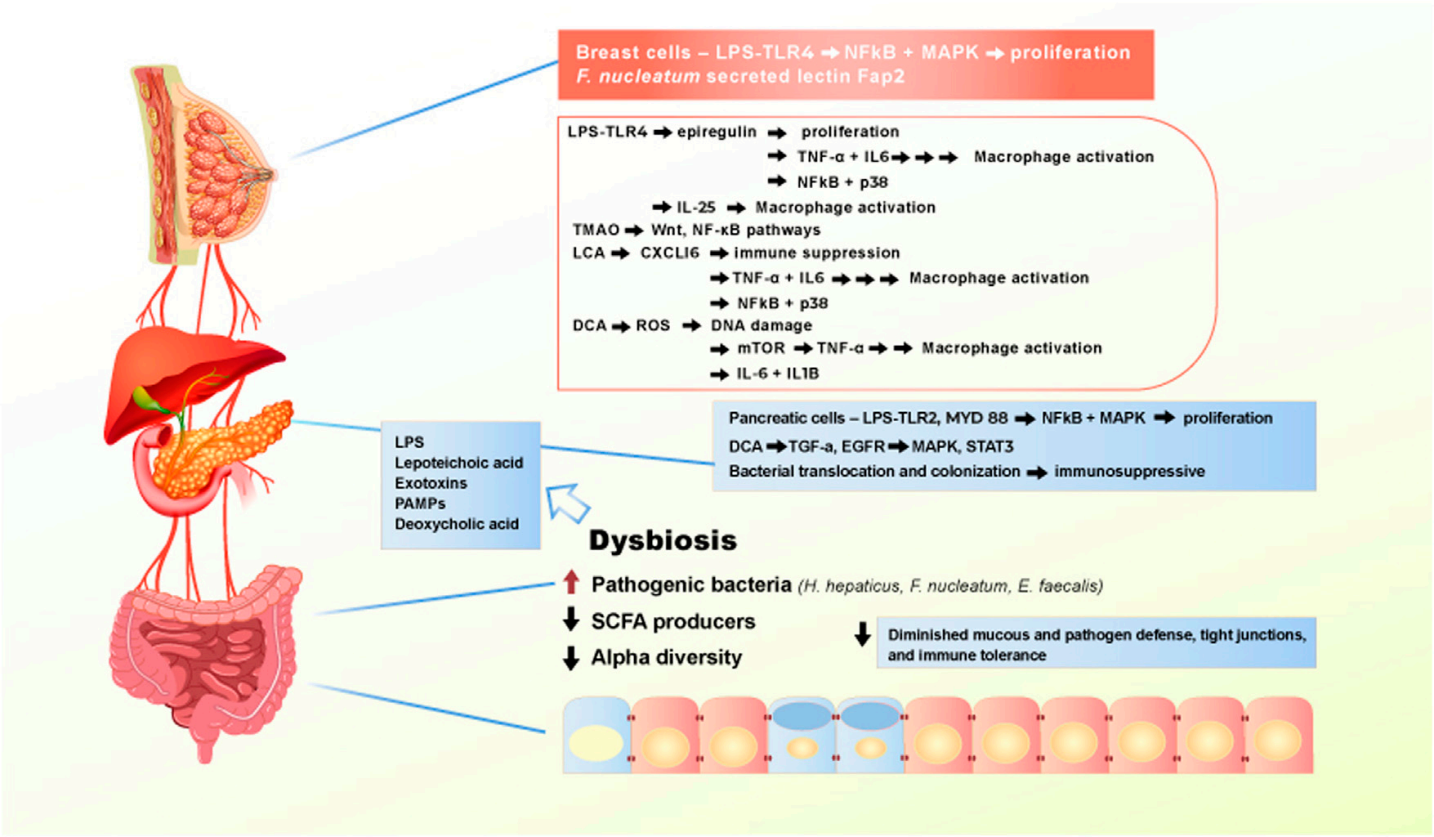
Effect of Western diet on the gut microbiome and extraintestinal pro-carcinogenic pathways. Adherence to Western diet promote the establishment of chronic dysbiosis in the gut, leading to translocation of pathogenic bacteria and toxic bacterial metabolites entering systemic circulation and reaching extraintestinal organs. Main drivers of the pro-carcinogenic and pro-inflammatory processes are LPS, lipoteichoic acid, numerous exotoxins, PAMPs, and deoxycholic acid. When bacterial metabolites reach liver, pancreas, and breast, they bind to receptors, such as TLR4 to activate signaling pathways that drives inflammation and proliferation.

Dysbiosis is implicated as a bridge between changing gut microbiome composition and the incipient manifestation of extraintestinal tumors. The microbial balance shifts away from commensal bacteria in the gut, creating a favorable environment for chronic inflammation as well as the suppression of immune surveillance (Zhou et al., 2020; Kovács et al., 2020). Intriguingly, it has been hypothesized that pathogenic bacteria, bacterial products, and metabolites escape into the systemic circulation via increased leakiness of tight junctions and contribute to promoting inflammatory pathways in other organs. This amounts to an organismal-level of carcinogenic circulatory signaling instigated by the diet-deregulated microbiome. Contemporary efforts in this field have examined the contribution of the gut metagenome to the development of conditions such breast, liver, and pancreatic cancers (Figure 3) (Chen et al., 2019; Jain et al., 2021).

Breast cancer is a multifactorial disease, aside from the rare minority of hereditary cases driven by BRCA1 and BRCA2 mutations, and familial predisposition involving genetic modifiers such as CHK2—that is initiated by a sequence of pro-carcinogenic events, including destabilized hormonal homeostasis. Growing incidence of obesity induced breast cancer, led to the acknowledgement of a gut-breast axis. Ley et al., have demonstrated that gut microbial composition differs between lean and obese people. The group has observed that Bacteroides phyla is significantly decreased in obese participants, supporting the evidence that gut microbiome mirrors the dietary and lifestyle choices (Ley et al., 2006). Moreover, Shively et al. designed a study focusing on the effect of Mediterranean versus Western diets on mammary gland microbiome of non-human primates. The group observed that upon administration of Mediterranean diet, *Lactobacillus* abundance increases in the mammary gland microbiome. Multiple studies have also shown altered gut microbiome in breast cancer patients as compared to healthy individuals (Shively et al., 2018; Iyengar et al., 2019; Klann et al., 2020; Yaghjyan et al., 2021). Nagrani et al., have demonstrated that obesity is a key risk factor in developing breast cancer, regardless of menopause manifestation and administration of hormonal therapy (Nagrani et al., 2016). The population-based study was performed between 2009 and 2013 amongst Mumbai female population, revealed the consistency of breast cancer diagnosis amongst women with higher BMI (Nagrani et al., 2016). Interestingly, Kabat et al. have conducted a 15-years prospective cohort study, focusing on the incidence of postmenopausal breast cancer and its association with obesity and metabolic syndrome (Kabat et al., 2017). Amongst 21,000 enrolled postmenopausal participants, 1,176 cases of invasive breast cancer were documented. The 15 years-follow up showed that obesity is the key risk factor of breast cancer manifestation, irrespective of metabolic dysregulation. Kabat et al. confirmed that the increase in BMI is directly proportional to the development of breast tumorigenesis (Kabat et al., 2017).

Parhi et al. showed that the increasing abundance of *F. nucleatum* positively correlates with metastatic progression of breast cancer in mice (Parhi et al., 2020). They showed that colonization of breast cancer cells is solely dependent on the *F. nucleatum* secreted lectin Fap2. Interestingly, inactivation of Fap2 significantly suppressed the tumor growth. Furthermore, metastatic progression of the cancer was facilitated by Fap2 and TIGIT binding, as this interaction results in abrogation of NK cells and tumor-infiltrating T-cells. Remarkably, breast cancer progression and speed of metastatic lesion formation was slowed down upon administration of antibiotic therapy (Parhi et al., 2020). This provides some evidence for the direct causal contribution of the gut microbiome to carcinogenesis beyond CRC, specifically to breast cancer progression.

Furthermore, Rao et al. have shown that chronic inflammation in the gut is a precursor of tumorigenesis in the mammary glands (Rao et al., 2006). They introduced *Helicobacter hepaticus* to female mice and observed the development of both mammary carcinoma and intestinal tumor. The main mechanism *H. hepaticus* utilizes to induce carcinogenesis was solely TNF-α dependent. By increasing the population of pro-inflammatory cytokines that enter the systemic circulation, *H. hepaticus* can establish the pro-inflammatory microenvironment in the breast tissues as well. This finding supports the claim that intestinal inflammation can result in systemic inflammation, causing neoplastic formation in distant organs (Rao et al., 2006). Rutkowski et al. showed that depletion of commensal bacteria in the gut increases the activity of TLR5, resulting in the production of pro-inflammatory cytokines such as IL-6 and elevation of the γδ T cell pool. This sequential cascade spreads inflammation and can fuel tumor microenvironment in the mammary tissues, resulting in tumor formation (Rutkowski et al., 2015)*.* Recently, Zhu et al., using shotgun metagenomic analysis of both premenopausal and postmenopausal breast cancer patients, have shown that breast cancer patients have a unique alteration of their gut microbiome in comparison to premenopausal healthy controls (Zhu et al., 2018). They demonstrated an enrichment of bacterial population involved in LPS biosynthesis, which is a stimulus for chronic intestinal inflammation (Hsu et al., 2011; Yin et al., 2016; Zhu et al., 2018). Taken together, these and other studies demonstrated that commensal bacteria are vitally important to maintaining immune homeostasis, not only locally in the gut microbiome, but also systemically in distant organs.

In the recent years, with the rising incidence of pancreatic cancer, the gut microbiome was explored as a potential driver of pancreatic malignancies. Immediate anatomical connection between pancreas and gastrointestinal system implicates a role of gut-pancreas axis in pathological conditions like type II diabetes, pancreatitis, and pancreatic ductal adenocarcinoma (PDAC). There is a unique alteration of gut microbiome between PDAC patients in comparison to healthy individuals (Pushalkar et al., 2018). Maekawa et al. have demonstrated that population of *Enterococcus faecalis*, a virulent bacterium that is more abundant in people adhering to the Western diet, is elevated in patients with pancreatic cancer, supporting the evidence of a potentially deleterious crosstalk between the gut microbiome and pancreas (Maekawa et al., 2018).

It is hypothesized that LPS translocated from the gut microbiome can cause high grade inflammation and tumorigenesis in the pancreas via the activation of MYD88 and TLR2. Interestingly, Nagathihalli et al. demonstrated that secondary bile acids, particularly deoxycholic acid (DCA) promote tumorigenesis in CRC and PDAC (Nagathihalli et al., 2014). It was observed that in pancreatic tissue, DCA modulate carcinogenic activity through TGF-α and EGFR interacting with pancreatic cells, further activating MAPK and STAT3 signaling pathways.

In mice and humans, there is a characteristic change in the microbiome signature associated with cancerous pancreas as compared to that in healthy pancreas. Pushalkar et al. have recently demonstrated that the microbiome in PDAC shifts from Bacteroidetes and Firmicutes to Proteobacteria, Actinobacteria and Fusobacteria, resulting in dysbiosis and increased bacterial translocation into the pancreatic duct (Pushalkar et al., 2018). Several studies have demonstrated that progressive carcinogenesis of PDAC is solely dependent on toll-like receptor (TLR) activation. Activation of the TLR family results in dramatic suppression of immune surveillance through increased pool of Th2-deviated CD4^+^ T cells and polarization of tumor-associated macrophages M1, leading to the rapid progression of PDA in mice. Removal of the microbiome-derived pathogens results in positive outcomes for PDAC by causing a reduction in myeloid-derived suppressor cells and an increase in M1 macrophage differentiation (Zambirinis et al., 2015; Pushalkar et al., 2018). In addition, emerging data indicates the mycobiome may play a significant role in pancreatic oncogenesis. Aykut et al. demonstrated that certain genera of endoluminal fungi promote the development of pancreatic oncogenesis in mice, whereas the fungal ablation was shown to be tumor-protective in this mouse model of pancreatic cancer (Aykut et al., 2019).

The role of the gut-liver axis has been extensively explored in the last decade, due to the increasing evidence of strong association between metabolic disease, obesity, adherence to a Western diet, and liver cancer. Although up to now the full spectrum of gut-liver crosstalk is yet to be determined, gut microbiome seems to add a tremendous contribution in the liver homeostasis.

Gut-liver axis and bi-directional metabolite translocation is thought to be maintained through biliary duct, portal vein and systemic circulation (Schwabe and Greten, 2020; George et al., 2021). Patients with hepatocellular carcinoma (HCC) have been shown to have reduced SCFA production and increased proinflammatory bacterial species (Muñoz et al., 2019; Suriguga et al., 2020). The disruption of gut barrier in events like dysbiosis and leaky gut, result in continuous secretion of microbial associated molecular patterns (MAMPs) such as LPS into the portal vein and systemic circulation. MAMPs act as ligand to TLR4 and other members of TLR family, initiating cascade of inflammatory reactions via activation of molecular pathways like Wnt and NF-κB, promoting production of inflammatory like cytokines IL-6 and TNF- α (Dapito et al., 2012). These TLR4 type receptors are found on multiple cell types in the liver, including hepatocytes, stellate cells, and Kupffer cells, that lead to changes in proliferation, fibrosis, and immune regulation (Yu and Schwabe, 2017; Yu et al., 2010). Fox et al. observed that upon *H. Hepaticus* infection, mice had increased promotion of tumorigenesis in the liver and lower bowel. The group observed activation of NF-κB pathway, which increased T helper 1 (Th1) immune response in both liver and colon. Moreover *H. Hepaticus* infection activated Wnt/β-catenin pathways, which led to cell proliferation as well as poor clearance of damaged hepatocytes (Fox et al., 2010).

Nutrients in food products can directly interact with gut microbiome, altering its composition. An emerging biomarker for some cancers is trimethylamine N-oxide (TMAO), a secondary metabolite produced upon red meat consumption. L-carnitine and choline from meat are metabolized to trimethylamine (TMA) by gut bacteria, this is absorbed and goes to the liver via the portal vein, where it is metabolized into TMAO. There are several lines of evidence linking elevated TMAO production with carcinogenesis and cardiovascular and other NCD (Liu et al., 2018; Zmora et al., 2019; Li et al., 2021a). It has been reported that TMAO production alters the population of the mucin-degrading bacteria *Akkermansia muciniphila* in the gut lumen, resulting in disruption of the protective mucous layer that covers intestinal epithelial cells (Ijssennagger et al., 2015; Zhu et al., 2016). TMAO was also found to be elevated in the serum of CRC patients, and the level of TMAO correlated inversely with patient prognosis, higher levels translating into less survival.

A recent population-based study on 671 patients with primary liver cancer (PLC), demonstrated that TMAO serum level was significantly elevated in patients with PLC compared to healthy controls (Liu et al., 2018). Interestingly, TMAO level was significantly decreased by reducing consumption of animal-based products (Oellgaard et al., 2017; Nowinski and Ufnal, 2018). A cohort study based in Finland showed that TMAO level positively associated with development of aggressive prostate cancer (Mondul et al., 2015). Additional epidemiological studies demonstrated the association of TMAO with liver cancer and CRC (Cho et al., 2017; Guertin et al., 2017). It is still unclear how exactly TMAO modulates carcinogenic processes. A possible mechanism that TMAO production enhances the activation of the Wnt and NF-κB signaling pathways, promoting a pro-inflammatory environment (Xu et al., 2015; Chan et al., 2019).

Bile acid homeostasis is also one of the key components of normal liver and intestine functioning. Bile acids that escape the absorption by the small intestine, travel to the colon, where they undergoes conversion into secondary bile acids. Interestingly secondary bile acid is increased in CRC and HCC patients, as well as the bacteria metabolizing the secondary bile acids (Modica et al., 2009). Large amounts of secondary bile acids can reach systemic circulation via portal vein, leading to liver inflammation (Wang et al., 1999; Zhou et al., 2020). The secondary bile acid deoxycholic acid can induce ROS mediated DNA damage and alter hepatic stellate cell phenotype resulting in IL-6 and IL-1beta production. One of the key components in bile acid homeostasis is farnesoid X receptor (FXR) (Yoshimoto et al., 2013). FXR belongs to the nuclear receptor family, that are mainly expressed in liver, kidneys, and intestine. Different types of bile acids, including taurocholic acid (TCA), deoxycholic acid (DCA) and cholic acid (CA), act as ligand for FXR, this interaction leads to the suppression of bile acid production. In cases of FXR deactivation, the bile homeostasis is disrupted resulting in continuous production of bile acids (Modica et al., 2009; Gonzalez et al., 2016).

Ma et al., has recently demonstrated that increase in natural killer T cells population inhibits the tumor progression in liver (Ma et al., 2018). It was observed that increase in Clostridium Cluster XIV abundance promoted secondary bile acid production, inhibited NKT cells, consequently leading to the progression of liver tumorigenesis. By administrating vancomycin antibiotic treatment researchers were able to deplete Gram-positive bacterial population in mice microbiome. Antibiotic treatment allowed reduced production of secondary bile acid and an increase in the translocation of CXCL16 ligand from the gut to the liver. CXCL16 is the key regulator of liver sinusoidal endothelial cells, by elevating the levels of CXCL16 researchers were able to improve the liver barrier from the gut circulated blood. This evidence supports the association between gut microbiome composition and liver homeostasis. Microbiome composition influences every organ and system in the entire body, however the majority of studies focused on specific microbes out of entire microbial populations, perhaps limiting the understanding of the microbial activity in certain diseases. Therefore, taking more systematic approach and trying to mimic entire gut microbiome signatures may unravel new avenues in cancer research.

## Alcohol—Molecular Mechanisms of Local and Systemic Effects

Alcohol use is common worldwide across most dietary patterns. Estimates from an international WHO survey indicate that the mean lifetime prevalence of alcohol use in all countries is 80%, with a range of 3.8–97.1%. The combined average population lifetime prevalence of alcohol use disorders is 8.6%, ranging from 0.7% in Iraq to 22.7% in Australia. As of 2016, the WHO estimated that 2.3 billion people were current drinkers and 283 million people (5.1% of adults) had alcohol use disorder. Since alcohol use is so common, most studies on the effect of specific diets on the intestinal microbiome likely include both alcohol and non-alcohol users. Approximately 4% of all cancers are caused by the overconsumption of alcohol, primarily including cancers of the upper aerodigestive tract, liver, colorectum, and breast. Alcohol has a myriad number of toxic and proinflammatory effects (Rumgay et al., 2021) that are beyond the scope of this review, we will focus here on microbiome-related changes.

Mouse models have revealed profound changes in intestinal barrier function induced by chronic alcohol use. The first changes observed were increased intestinal bacterial overgrowth and altered microbiome profiles (Yan et al., 2011). This is also associated with decreased expression of intestinal bactericidal c-type lectins Reg3b and Reg3g, which help regulate luminal bacteria (Yan et al., 2011). Interestingly, treatment with prebiotics could help restore Reg3g levels, reduce bacterial overgrowth, and decrease steatohepatitis. Alcohol use for 8 weeks results in reduced intestinal barrier function and increased intestinal inflammation characterized by an increased number of inflammatory cells expressing tumor necrosis factor alpha (TNF-a) (Chen et al., 2015). This is accompanied by translocation of microbial products to the liver and increased steatohepatitis.

**FIGURE 4:**
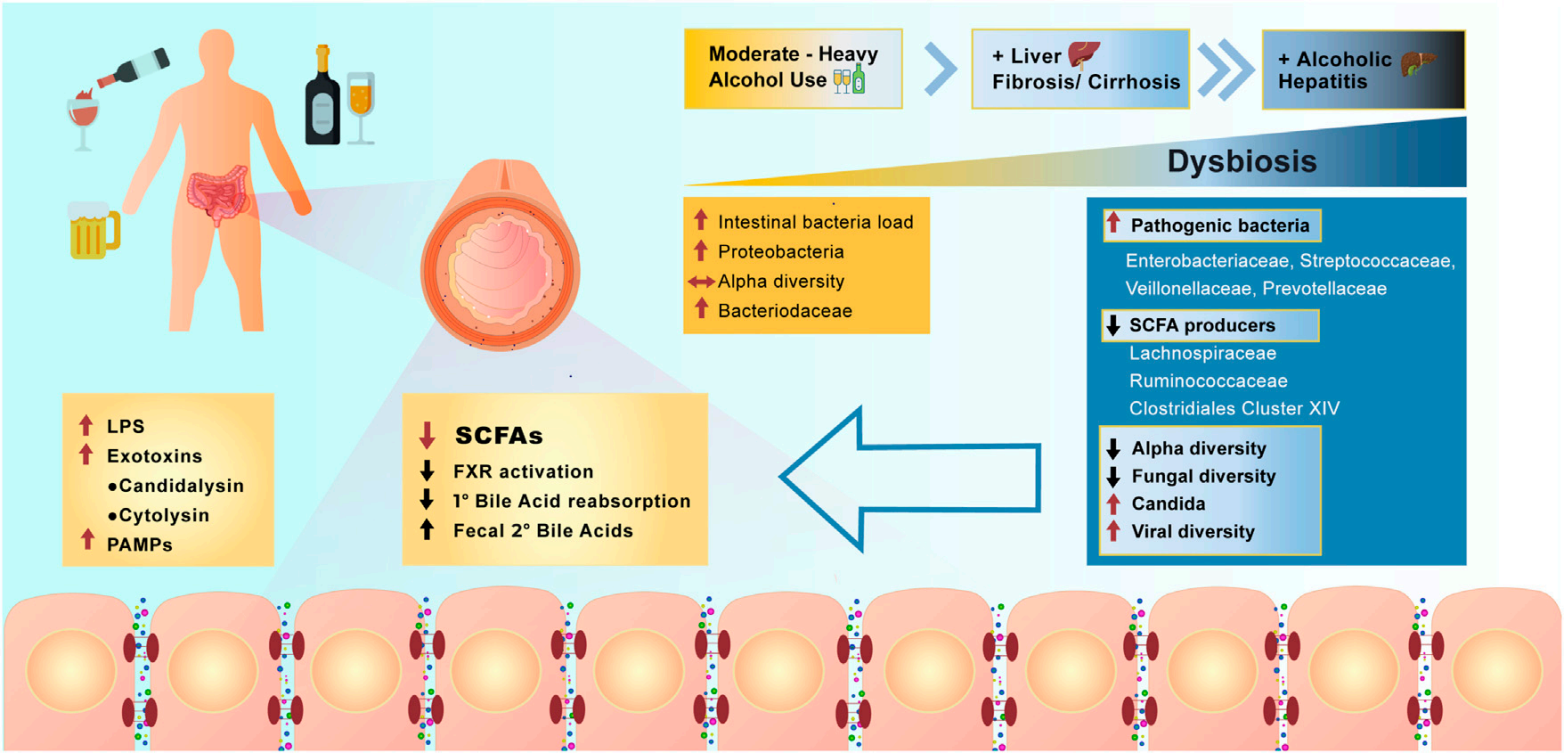
Effect of alcohol overconsumption on the gut microbiome and intestinal homeostasis. Over consumption of alcohol positively correlates with the severity of manifested dysbiosis in the lower bowel and occurrence liver diseases. Dysbiosis resulted from alcohol overconsumption, leads to the increase in abundance of pathogenic bacteria, increase in viral diversity, production of LPS, secondary bile acids and activation of farnesoid X receptor (FXR). Alcohol overconsumption also downregulates production of SCFAs, results in decreased alpha and fungal diversity and decrease reabsorption of primary bile acids (PAMPs = pathogen associated molecular patterns).

Studies of the effect of alcohol on the microbiome in humans have focused on subjects with moderate to heavy alcohol use, with or without concomitant liver disease (Figure 4). With moderate alcohol and before the development of significant liver disease, initial changes include an increase in the numbers of bacteria cultured from the small intestine, similar to what is observed in mouse models of alcohol feeding. Further studies have shown additional qualitative changes in 16S rRNA analysis of bacterial species, including a relative increase in *Proteobacteria* and a decrease in *Bacteriodaceae*, however overall alpha diversity remains stable. Increasing burden of alcohol use with concomitant liver fibrosis or cirrhosis results in further changes in the microbiome, resulting in major metabolic consequences affecting intestinal cells and systemic pathways (Fairfield and Schnabl, 2021). Analysis of stool specimens of patients with cirrhosis and alcoholic hepatitis demonstrate a decrease in SCFA producing bacteria, with an increase in potentially pathogenic bacteria, such as *Enterovaeteriaceae, Streptococcaceae, Veillonellaceae,* and *Prevontellaceae*. There is an overall decrease in alpha diversity in luminal bacteria. With the most severe form of alcoholism and liver disease, alcoholic cirrhosis and alcoholic hepatitis, further reductions in fungal diversity with an overgrowth of *Candida* species has been demonstrated (Yang et al., 2017a). In patients with alcoholic hepatitis these changes are accompanied by an increase in the viral microbiome diversity compared with subjects with alcohol use disorder and non-alcoholic controls (Jiang et al., 2020). The viral diversity is in large part related to increases in *Escherichia*-, *Enterobacteria*-, and *Enterococcus* bacteriophages and an increase in an often underappreciated non-bacterial component of the microbiome—mammalian viruses, such as *Parvoviridae* and *Herpesviridae*. In contrast, decreased viral diversity was found in patients with non-alcoholic fatty liver disease (NAFLD) and non-alcoholic steatohepatitis (NASH)-cirrhosis, indicating that increased fecal viral diversity is unique to the effects of alcohol and/or alcohol-related liver disease (Lang et al., 2020).

The changes in the microbiome associated with heavy alcohol use have been correlated with worse medical outcomes in individual patients. Lang et al., have shown that patients with alcoholic cirrhosis have reduced fungal diversity and an overgrowth of *Candida* species compared with healthy individuals and non-alcohol-related cirrhosis. In the alcoholic cirrhosis subjects, this is accompanied by increased systemic fungal antigens and higher risk for death (Lang et al., 2020). These findings are duplicated in mouse models of alcoholic hepatitis with demonstration of increased circulating fungal antigens and increased liver inflammation via stimulation of C-type lectin like receptor CLEC7A on hepatic Kupffer cells resulting in cytokine IL-1b release (Yang et al., 2017b; Yang et al., 2019). Furthermore, qualitative differences in fecal viral taxa are associated with worse 90-days survival in patients with alcoholic hepatitis (Jiang et al., 2020). Currently there is much interest in whether interventions targeting the specific fungal and virome changes associated with poor prognosis can improve patient outcomes. To demonstrate the feasibility of this approach, Duan et al. showed that patients with alcoholic hepatitis have increased abundance of cytolysin-positive *Enterococcus faecalis*, which correlates with increased mortality (Duan et al., 2019). Germ free mice colonized with cytolysin-positive *E. faecalis* from patients with alcoholic hepatitis had worse liver disease in a model of alcohol induced liver disease. Mice were treated with bacteriophages that target and destroy cytolysin-positive *E. faecalis* and they showed that this could ameliorate alcohol induced liver disease in mice. Further studies targeting specific pathogenic bacteria implicated by metagenomics in human diseases are needed.

## The Next Step—Genetic Epidemiology and Population Genomics Could Reveal Possible Links Between Host Genetics and Microbiome

In addition to diet, host genetics is an important factor that influences the gut microbiome, predisposing individuals to microbiome-modulated pathologies. A genetic bias for microbiome composition is suggested by studies of association and heritability of certain bacterial species in related individuals. Monozygotic twins have higher correlation of microbiome signatures than dizygotic twins (Goodrich et al., 2016). Host genetic factors can influence the microbiome in mice (Benson et al., 2010). Genome-Wide Association Studies (GWAS) of the microbiome have revealed only limited links to host genetics. The detection of only a small number of associated loci is primarily due to inter-individual variability, microbiome heterogeneity, and potential overshadowing of host-genetics factors by the contributions of the diet, environment, and lifestyle to microbiome composition (Kurilshikov et al., 2021). While the link between host genetics and microbiome diversity may be weak, genetics can modulate specific microbiome-diet, microbiome-environment, and microbiome-lifestyle interactions in ways that lead to specific disease outcomes which are traceable to specific molecular interactions between host- and microbiome-derived components. Well-controlled studies, especially those utilizing homogenous populations, may be able to reveal these impacts more easily. Despite these limitations, meta-analyses of GWAS have proven effective in identifying specific molecular mechanisms connecting the microbiome to numerous diseases, including cancer. In particular, TMAO, the microbiome-generated metabolite of red meat and fat previously connected with cardiometabolic risks, was additionally linked to CRC risk by this approach (Xu et al., 2015). Aside from colon cancer, other cancers, including bladder and prostate cancer, show clear associations with the microbiome in GWAS. These signals were identified through correlating significant disease-associated genetic variants from GWAS with microbiome data. The putative causative relationships do not just go “From microbiome to cancer”, as they also go the other way around: “From cancer to microbiome.” Atrial fibrillation, chronic kidney disease, and prostate cancer, as predicted by host genetics, have potential causal effects on the abundance of specific gut microbiome components (Xu et al., 2015). This bidirectionality of the cancer-microbiome axis is important to take into account, because—while the idea that the microbiome can regulate, or influence disease pathogenesis is more intuitive and widely accepted—it means that a disease state can also influence the microbiome (with potential downstream feedback effects on the disease).

Given the abundance of long non-coding RNA (lncRNA) genes compared to conventional protein-coding genes in humans, it is expected that GWAS-driven interrogation of the microbiome-disease connection will uncover lncRNA contributors to this class of regulatory phenomena. Accounting for well over half of human genes, lncRNAs are now increasingly understood to be fundamental and essential to all normal cellular and developmental processes, as well as all human diseases, in which they have been examined (Hong et al., 2020). There is already a precedent: non-coding genetic variants from GWAS, including those in a lncRNA gene, have been associated with defined phyla within the microbiome (Hughes et al., 2020). Mouse microbiome models demonstrate that lncRNAs can be microbiome targets in regulatory networks, building a case for microbiome-lncRNA interactions, with functions mediated in part through the role of lncRNA in immunity (Allen and Sears, 2019) (Hong et al., 2020).

A recent study of 33 CRC patients has revealed a connection between the high abundance of *F. nucleatum* and elevated glucose metabolism: a spectrum of upregulated lncRNA in CRC cells infected with *F. nucleatum* that had increased glycolysis rate compared to uninfected CRC cells. The ENO1-IT1 lncRNA, a positive *cis*-regulator of the pro-oncogenic, pro-glycolysis ENO1 gene is the most overexpressed lncRNA correlated with glycolysis. Upon *F. nucleatum* infection, the transcription factor SP1 promotes the expression of ENO1-IT1 lncRNA through the ENO1 signaling pathway. ENO1-IT1 eventually forms a complex with KAT7 guide protein that increases the histone acetylation leading to the CRC progression (Hong et al., 2020). Chen at el., recently observed that *F. nucleatum* directly drives the overexpression of long non-coding RNA Keratin7-antisense (KRT7-AS) and Keratin7 (KRT7) in human CRC cells. *In vitro* and *in vivo* analysis showed that *F. nucleatum* activates the NF-κB signaling pathway, which upregulates KRT7-AS (a positive regulator of CRC metastasis) which in turn, serves as an activator of KRT7, stimulating cell migration (Figure 2) (Chen et al., 2020b).

Beyond specific case studies of human microbiome-lncRNA interactions, mouse models suggest that whole-genome lncRNA expression profiles distinguish between mouse gut microbiomes better than protein-coding gene mRNA signatures (Liang et al., 2015)– a key finding that cements the importance of lncRNAs as key components of the microbiome-disease axis and justifies further investigation of the molecular and functional underpinnings of their global relationship to the microbiome-cancer interface. The fundamental contribution of lncRNAs to cancer is, by now firmly established (Allen and Sears, 2019). Oncogenic lncRNAs have an array of roles linked to microbiome-related metabolites in cancer. *Fusobacterium* promotes the EMT transition in cancer through a long non-coding RNA gene that also serves as a microRNA host gene (Zhang et al., 2020). Nevertheless, rather than serving solely as drivers of disease progression, ncRNAs, if arising from disease-protective microbiomes and/or under disease-risk-reducing dietary conditions, may have protective roles. This possibility is consistent with the observed downregulation of several microRNAs in *F. nucleatum*-rich tumors in patients with recurrent CRC (Allen and Sears, 2019).

Concordant with this hypothesis, 30 lncRNAs have recently been highlighted to be involved in the inhibition of CRC progression by sodium butyrate (NaB) (Xiao et al., 2018). Butyrate-responsive lncRNAs have also been identified in a lung cancer model (Xi et al., 2021). RAD51-AS1, the endogenous antisense transcript that overlaps and modulates the tumor suppressor RAD51, regulates lactate in CRC (Li et al., 2021b). Although direct links with the microbiome have not yet been proven for these lncRNAs, it is reasonable to posit that, because the microbiome is tightly coupled to SCFA and lactate metabolism, so are these lncRNAs, given their direct interactions with these metabolites and pathways.

The specific molecular mechanisms that are responsible for, and mediate, the existence of multiple directional nodes joining microbiome-derived and host RNA-derived edges in the microbiome-cancer regulatory network should be characterized in future work, so that they can be rationally targeted for therapeutics.

## Future Directions

In this review we discussed recent studies that highlight role of microbiome, in particular abundance of certain pathogenic bacteria including *F. nucleatum* and *pks* ^*+*^ *E. coli*, that drive the process of carcinogenesis through induction of DNA damage, overexpression of oncogenes like Myc and triggering production of highly pro-inflammatory agents like IL-6. This review allows the reader to clearly grasp the main metabolic and molecular events that lead towards intestinal and systemic carcinogenesis upon adherence to low fiber, high fat Western dietary pattern. To compare, this review describes recent studies that uniformly demonstrate plant-based diet as a protective factor from set of metabolic conditions including obesity and from various types of cancer, including CRC. Due to the limitations of space the review is primarily focused on human studies, and extensive review of many animal studies related to microbiome and cancer are not included. The protective properties of plant-based diet are associated with the increased production of SCFAs by the commensal bacterial in the gut. The SCFAs are one of the key regulators of immune tolerance, improved gut barrier junctions, and the intestinal clearance. The depletion of SCFA producers or SCFA receptors result in adverse effects including high-grade inflammation and poor cancer prognosis. In contrast, elevating the abundance of SCFA producing bacteria in the gut microbiome through the dietary intervention, results in downregulation of inflammation and inhibition of tumor microenvironment. Although microbiome-cancer axis has been extensively studied in the past three decades, there is tremendous amount of vital information that is yet to be discovered. This review indicates that diet is the major regulator of gut microbiome and can act as a first line preventive measure from developing carcinogenic conditions. Moreover, major bacteria and metabolites that are associated with detrimental effects of Western diet on intestinal and systemic homeostasis that were discussed in this review, can serve as a potential therapeutic targets in a variety of diseases, especially cancer. Diet, as a key component of our life should not be regarded only from the nutritional point of view. Being selective, consistent, and conscious about the personal diet may prevent a one’s spectrum of health conditions and improve the quality of life. Growing numbers of studies are focusing on manipulation of gut bacterial composition to enhance the efficacy of anti-cancer therapies. Several recent studies have shown significant improvement in anti-CTL4 and anti-PD1 based therapies, via alternating gut microbiome composition towards certain commensal bacterial species, including *Bifidobacterium species* (Sivan et al., 2015; Vetizou et al., 2015). Using dietary intervention, to enhance certain commensal bacteria population, can be a supportive measure in vast array of diseases, including cancer. Personalized targeted microbial therapy is one of the most promising novel therapeutics. Currently used antibiotics are of a broad spectrum and are detrimental for both commensal and pathogenic bacteria in the gut microbiome. In situations like cancer, antibiotic established dysbiosis is a potential threat to the successful cancer therapy. Using novel bioinformatic tools and established metagenomic and metabolomic data, we can now begin to create a more personalized approach to cancer therapy and prevention. Soon we will be able to monitor and shift microbial signatures to high SCFA and anti-inflammatory metabolite producers, and target specific harmful bacteria using selective antibiotics, bacteriophages, or competitor probiotic species. Numerous randomized trials of specific gut microbiome therapeutics will be needed to expand and prove these concepts.
