# Supplementary material for: Dietary Patterns and Associated Microbiome Changes that Promote Oncogenesis
Source: Front Cell Dev Biol. 2021 Nov 12;9:725821. doi: 10.3389/fcell.2021.725821 (PMC8633417; doi:10.3389/fcell.2021.725821)
Supplement: Supplementary file 1 [file Table1.pdf]

Table S1: Different dietary indices, their scoring methodologies and examples of correlated health outcomes

Diet Indices and description	Scoring		Correlation with health outcomes
	Methodology	Range	
**alternate Mediterranean Diet Index (aMED)**<br>***Measures adherence to a Mediterranean diet* [1].**	1 point each for greater than median consumption of vegetables, legumes, fruit, nuts, whole grains, monounsaturated fats and fish and 1 point each for less than median consumption of red and processed meats and alcohol	0 to 9	Top 20% scorers have lower inflammation and endothelial dysfunction than bottom 20% scorers [1]<br>Top 20% scorers have 16% lower risks of T2D than bottom 20% [2].<br>Higher adherence is inversely correlated with colorectal cancer risk in men but not women [3][14]
**Alternate Healthy**<br>**Eating Index (aHEI)**<br>***Measures conformance to the 2010 Dietary Guidelines for Americans* [1; 4].**	10 points for appropriate consumption of Vegetables, Fruit, Nuts and soy, higher ratio of white to red meat, cereal fiber, polyunsaturated fat, multivitamin, and alcohol and 10 points for diversity in foods consumed	0 to 100	Top 20% scorers have lower inflammation and endothelial dysfunction than bottom 20% scorers [1].<br>Higher adherence is inversely correlated with colorectal cancer risk in men but not women [3][14]
**US Healthy Food Diversity index (HFD)**<br>***Measures dietary variety, dietary quality and portion***<br>***according to the 2010 Dietary Guidelines for Americans* [5].**	Sum of scores calculated for each individual food item, based on the following equation:<br>US HFD Index = $(1-\sum s_i^2)*hv$<br>Where s_i is share of food group i, and hv is health factor of food.	0 to 1	Top third scorers had 21% lower odds of MetS than bottom third scores [5].
**DASH score**<br>***Measures adherence to DASH guidelines (see Table 1) [1].***	Sum of scores calculated for following 8 food groups (ranging from 1 to 5 based on consumption): high intake of fruits, vegetables, nuts and legumes, low-fat dairy products, and whole grains; low intake of sodium, sweetened beverages, and red and processed meats.	8 to 40	Top 20% scorers have 29% lower risks of T2D than bottom 20% [2].<br>Women who score in top 20% have 76% less risk of coronary heart disease than bottom 20% [6].<br>Higher adherence is inversely correlated with colorectal cancer risk in men but not women [3][14]

**PDI (plant-based diet index)**<br><br>***Quantifies consumption of all plant-based foods in diet* [7].**	Positive scores (1 to 5, with 5 for highest consumption) are given to plant foods and similarly reverse scores to animal foods.	17 to 85	Top 25% scores have 67% lower odds of breast cancer than those in bottom 25% [8].
**Healthful PDI (hPDI)**<br><br>***Quantifies consumption of healthy plant-based foods in diet* [7; 9].**	Same as for PDI, but positive scores are only given to healthy plant foods (whole grains, fruits/vegetables, nuts/legumes, oils, tea/coffee), and less-healthy plant foods and animal foods are given reverse scores.	17 to 85	Higher scorers have 36% less risk of developing breast cancer [8].<br><br>This dietary pattern is associated with a reduced risk of all colorectal cancers [10]
**Unhealthful PDI (uPDI)**<br><br>***Quantifies consumption of unhealthy plant-based foods in diet* [7; 9].**	Same as for hPDI, but positive scores are given to less-healthy plant foods ((juices/sweetened beverages, refined grains, potatoes/fries, sweets) and reverse scores to animal and healthy plant foods.	17 to 85	Top 20% have 50% higher risk of developing MetS [9].<br><br>Women with top 25% scores are 2.23 times more likely to have breast cancer than the bottom 25% scorers [8].
**Dietary inflammatory index (DII)**<br><br>***Calculates inflammatory potential of a diet [11]***	31 macro- and micro-nutrients are weighted based on consumption and multiplied by the inflammatory potential of each item	−8.87 to +7.98	Higher scores are 40% more likely to have colorectal cancer [12] [11]<br><br>Scores greater than -1.77 are 3.5 times more likely to have gastric cancer [13]

References

[1] T.T. Fung, M.L. McCullough, P.K. Newby, J.E. Manson, J.B. Meigs, N. Rifai, W.C. Willett, and F.B. Hu, Diet-quality scores and plasma concentrations of markers of inflammation and endothelial dysfunction. Am J Clin Nutr 82 (2005) 163-73.

[2] G.C. Chen, W.P. Koh, N. Neelakantan, J.M. Yuan, L.Q. Qin, and R.M. van Dam, Diet Quality Indices and Risk of Type 2 Diabetes Mellitus: The Singapore Chinese Health Study. Am J Epidemiol 187 (2018) 2651-2661.

[3] J. Petimar, S.A. Smith-Warner, T.T. Fung, B. Rosner, A.T. Chan, F.B. Hu, E.L. Giovannucci, and F.K. Tabung, Recommendation-based dietary indexes and risk of colorectal cancer in the Nurses' Health Study and Health Professionals Follow-up Study. Am J Clin Nutr 108 (2018) 1092-1103.

[4] P.M. Guenther, K.O. Casavale, J. Reedy, S.I. Kirkpatrick, H.A. Hiza, K.J. Kuczynski, L.L. Kahle, and S.M. Krebs-Smith, Update of the Healthy Eating Index: HEI-2010. J Acad Nutr Diet 113 (2013) 569-80.

[5] M. Vadiveloo, L.B. Dixon, T. Mijanovich, B. Elbel, and N. Parekh, Development and evaluation of the US Healthy Food Diversity index. Br J Nutr 112 (2014) 1562-74.

[6] T.T. Fung, S.E. Chiuve, M.L. McCullough, K.M. Rexrode, G. Logroscino, and F.B. Hu, Adherence to a DASH-style diet and risk of coronary heart disease and stroke in women. Arch Intern Med 168 (2008) 713-20.

[7] A. Satija, S.N. Bhupathiraju, D. Spiegelman, S.E. Chiuve, J.E. Manson, W. Willett, K.M. Rexrode, E.B. Rimm, and F.B. Hu, Healthful and Unhealthful Plant-Based Diets and the Risk of Coronary Heart Disease in U.S. Adults. J Am Coll Cardiol 70 (2017) 411-422.

[8] S. Rigi, S.M. Mousavi, S. Benisi-Kohansal, L. Azadbakht, and A. Esmaillzadeh, The association between plant-based dietary patterns and risk of breast cancer: a case-control study. Sci Rep 11 (2021) 3391.

[9] H. Kim, K. Lee, C.M. Rebholz, and J. Kim, Plant-based diets and incident metabolic syndrome: Results from a South Korean prospective cohort study. PLoS Med 17 (2020) e1003371.

[10] M.J. Orlich, P.N. Singh, J. Sabate, J. Fan, L. Sveen, H. Bennett, S.F. Knutsen, W.L. Beeson, K. Jaceldo-Siegl, T.L. Butler, R.P. Herring, and G.E. Fraser, Vegetarian dietary patterns and the risk of colorectal cancers. JAMA Intern Med 175 (2015) 767-76.

[11] N. Shivappa, S.E. Steck, T.G. Hurley, J.R. Hussey, and J.R. Hebert, Designing and developing a literature-derived, population-based dietary inflammatory index. Public Health Nutr 17 (2014) 1689-96.

[12] N. Shivappa, J. Godos, J.R. Hebert, M.D. Wirth, G. Piuri, A.F. Speciani, and G. Grosso, Dietary Inflammatory Index and Colorectal Cancer Risk-A Meta-Analysis. Nutrients 9 (2017).

[13] F. Vahid, N. Shivappa, Z. Faghfoori, A. Khodabakhshi, F. Zayeri, J.R. Hebert, and S.H. Davoodi, Validation of a Dietary Inflammatory Index (DII) and Association with Risk of Gastric Cancer: a Case-Control Study. Asian Pac J Cancer Prev 19 (2018) 1471-1477.

[14] Torres Stone R, Waring M, Cutrona S, Kiefe C, Allison J, Doubeni C. The association of dietary quality with colorectal cancer among normal weight, overweight and obese men and women: a prospective longitudinal study in the USA. 2021.
